# Landscape-scale accessibility of livestock to tigers: implications of spatial grain for modeling predation risk to mitigate human–carnivore conflict

**DOI:** 10.1002/ece3.1440

**Published:** 2015-03-02

**Authors:** Jennifer R B Miller, Yadvendradev V Jhala, Jyotirmay Jena, Oswald J Schmitz

**Affiliations:** 1 School of Forestry & Environmental Studies, Yale UniversityNew Haven, Connecticut, 06511; 2 Wildlife Institute of IndiaDehradun, Uttarakhand, 248001, India; 3 Satpuda Maikal Landscape Programme, WWF-IndiaMandla, Madhya Pradesh, 481661, India

**Keywords:** Carnivore conservation, human–wildlife conflict, India, livestock depredation, predation risk modeling, resource selection function

## Abstract

Innovative conservation tools are greatly needed to reduce livelihood losses and wildlife declines resulting from human–carnivore conflict. Spatial risk modeling is an emerging method for assessing the spatial patterns of predator–prey interactions, with applications for mitigating carnivore attacks on livestock. Large carnivores that ambush prey attack and kill over small areas, requiring models at fine spatial grains to predict livestock depredation hot spots. To detect the best resolution for predicting where carnivores access livestock, we examined the spatial attributes associated with livestock killed by tigers in Kanha Tiger Reserve, India, using risk models generated at 20, 100, and 200-m spatial grains. We analyzed land-use, human presence, and vegetation structure variables at 138 kill sites and 439 random sites to identify key landscape attributes where livestock were vulnerable to tigers. Land-use and human presence variables contributed strongly to predation risk models, with most variables showing high relative importance (≥0.85) at all spatial grains. The risk of a tiger killing livestock increased near dense forests and near the boundary of the park core zone where human presence is restricted. Risk was nonlinearly related to human infrastructure and open vegetation, with the greatest risk occurring 1.2 km from roads, 1.1 km from villages, and 8.0 km from scrubland. Kill sites were characterized by denser, patchier, and more complex vegetation with lower visibility than random sites. Risk maps revealed high-risk hot spots inside of the core zone boundary and in several patches in the human-dominated buffer zone. Validation against known kills revealed predictive accuracy for only the 20 m model, the resolution best representing the kill stage of hunting for large carnivores that ambush prey, like the tiger. Results demonstrate that risk models developed at fine spatial grains can offer accurate guidance on landscape attributes livestock should avoid to minimize human–carnivore conflict.

## Introduction

Many large carnivores remain perilously close to extinction despite concerted conservation efforts (Treves and Karanth 2003; Walston et al. 2010; Ripple et al. 2014). Continued human rural population growth, habitat degradation, and wild prey depletion have created fragmented, resource-limited landscapes for carnivores. Inevitably, carnivores seek alternative prey, leading to livestock losses and consequentially retaliatory killing by livestock owners (Treves and Karanth 2003; Dinerstein et al. 2007). One of the most time- and cost-efficient methods for reducing livestock losses is to avoid grazing domestic animals in areas where they are highly vulnerable to carnivore attacks (Treves et al. 2011). Thus, identifying the landscape features that facilitate predator access to prey and increase capture success can offer valuable insights to help livestock owners avoid losses by directing livestock into lower-risk areas where animals are less likely to be killed.

Carnivore species hunt repeatedly in areas characterized by a similar combination of landscape features where they can most easily access and kill prey (Hopcraft et al. 2005; Laundré et al. 2009). For large carnivores, these landscape features include a mix of land uses, vegetation structure, human activities, and prey densities (Table1; Gorini et al. 2012). Prey vulnerability to carnivores is likewise shaped by prey foraging patterns, habitat preferences, and antipredator behavioral responses, such that prey favor areas where they are less accessible to predation (Brown et al. 1999). Carnivores with similar traits, such as hunting tactic (e.g., ambush or active) and habitat domain (i.e., how a carnivore uses space and habitat within its home range), often hunt and kill prey with respect to similar landscape attributes. This causes prey to exhibit consistent predator risk responses, creating a tractable spatial distribution of predator–prey interactions often called the “landscape of fear” (Preisser et al. 2007; Laundré et al. 2010; Miller et al. 2014).

**Table 1:** Predictor variables used in the study, showing the data source, spatial grain, and evidence of variable importance for livestock depredation by large *Felidae* carnivores that ambush prey, especially tigers

Category	Predictor variable (unit)	Data source (spatial grain of raster)	Evidence of effect on predation risk
Human presence	Distance to road (m)	Survey of India topo maps from 1978, 1979, 1983, and 1984	Increased risk farther from roads^1^
	Distance to village (m)	Kanha Tiger Reserve Forest Department	Increased risk of farther from villages^1^
	Distance to core (m)		Increased risk closer to core^2^
Land use	Distance to nonforest (m)	Forest Survey of India State of the Forests 2009 (24 m)	Decreased risk in open forest^1^, ^2^, ^3^, ^4^, ^5^; agriculture poor habitat for tigers^6^
	Distance to scrubland (m)		Decreased risk in open forest^1^, ^2^, ^3^, ^4^, ^5^; less suitable habitat for tigers^6^
	Distance to moderately dense forest (m)		Increased risk in dense forest^1^, ^2^, ^3^, ^4^, ^5^; high suitable habitat for tigers^6^; common habitat type for tigers killing prey^7^
	Distance to very dense forest (m)		Increased risk in dense forest^1^, ^2^, ^3^, ^4^, ^5^; high habitat suitability for tigers^6^; common habitat type for tigers killing prey^7^
Vegetation structure	Visibility (m)		Increased risk with decreased visibility^4^, ^8^ but increasing vegetation cover^1^, ^2^, ^3^
	Shrub height (m)		Increased risk with increasing vegetation cover^1^, ^2^, ^3^
	Shrub cover (%)		Increased risk with greater vegetation cover^1^, ^2^, ^3^
	Shrub patchiness (%)		Increased risk with increasing vegetation cover^1^, ^2^, ^3^

1 Soh et al. 2014

2 Karanth et al. 2012

3 Kissling et al. 2009

4 Shrader et al. 2008

5 Valeix et al. 2009

6 Seidensticker 1976

7 Karanth and Sunquist 2000

8 Balme et al. 2007.

Identifying high-risk areas requires distinguishing between where prey are available versus *accessible* to carnivores (Trainor and Schmitz 2014). The mere presence of prey on the landscape (availability) is not sufficient to guarantee predator hunting success. Factors such as prey antipredator behavior and predator hunting tactics influence the locations where predators can successfully capture prey (accessibility). When selecting hunting sites, many large carnivores prioritize prey catchability as much as or more than prey abundance (Hopcraft et al. 2005; Holmes and Laundré 2006; Balme et al. 2007; Fuller et al. 2007; Laundré et al. 2009). Thus, analyses that combine spatial information on predator–prey interactions with an understanding of species abundance and distribution can offer much needed insight to characterize prey accessibility and spatial risk (Trainor and Schmitz 2014).

Such analyses are traditionally performed with resource selection functions (RSFs) that relate spatial environmental data (e.g., habitat features, topography) to the locations of predators and prey (Johnson et al. 2006; Gorini et al. 2012). Predation risk modeling enlists RSFs to focus on the spatial distribution of predator–prey interactions. Such modeling predicts the probability of a carnivore attacking prey by relating the environmental features at interaction sites (determined by encounters or kills) across landscapes with the number of interaction events at those sites relative to random sites representing landscape availability (Johnson et al. 2006; Treves et al. 2011). When models are applied to identify landscape locations of predator–livestock interactions (Treves et al. 2011), the insights can help to mitigate livestock depredation and thereby support large carnivore conservation by reducing retaliatory killing of carnivores by livestock owners (Gervasi et al. 2013; Soh et al. 2014).

The landscape features that best determine prey accessibility can vary over different stages of the hunt. This is because the heterogeneity of these features changes with the size of the area (spatial grain or the smallest unit of study, i.e., pixel or resolution) over which carnivore species make decisions (Gorini et al. 2012; Wilmers et al. 2013; Trainor and Schmitz 2014). The first few stages of the hunt – searching and encountering – may occur over a broad region of a large carnivore's home range, spanning square kilometers, whereas the final stages of greatest predation risk – attacking, killing, and consuming – occur over smaller areas, ranging square meters (Gorini et al. 2012). As the spatial scale of predator and prey decisions changes throughout the hunting process, different landscape attributes may determine whether and how carnivores access their prey and hence the resulting probability of predation (Hebblewhite et al. 2005; Hilborn et al. 2012). For example, the likelihood that elk and wolves encounter each other across a landscape was most influenced by topography, vegetation type, and resource selection by the carnivore; but once encountered, the final stages of hunting and hunting success were primarily affected by vegetation type alone (Atwood et al. 2009).

Mitigating human–carnivore conflict requires identifying the landscape features that reduce livestock accessibility and risk of death. As a case example of this process, we investigated tiger (*Panthera tigris*) depredation on livestock. Tiger depredation causes annual household income losses of up to 80% (Madhusudan 2003). This has prompted retaliatory killing of 1–22 tigers per year over the past two decades in many range countries (Inskip and Zimmermann 2009). With fewer than 3500 tigers left in the wild, developing risk maps to assist in reducing conflict may play an important role in helping to stem the species’ decline (Walston et al. 2010).

We provide insight into determinants of livestock accessibility to tigers, and hence depredation risk, by (1) developing a statistical model for predicting the spatial distribution of livestock kills by tigers; (2) identifying landscape characteristics associated with elevated vulnerability of livestock; and (3) identifying the spatial grain that best describes the risk of a kill. We map predictions of risk across the landscape to show how to visualize spatial hot spots of livestock kills (and hence hot spots of human–carnivore conflict) as well as validate predictions using an independent dataset of geospatial locations of livestock kills. We conclude by offering practical advice for the use of predation risk models in the conservation and management of large carnivore species.

## Materials and Methods

### Study area

**Figure 1:**
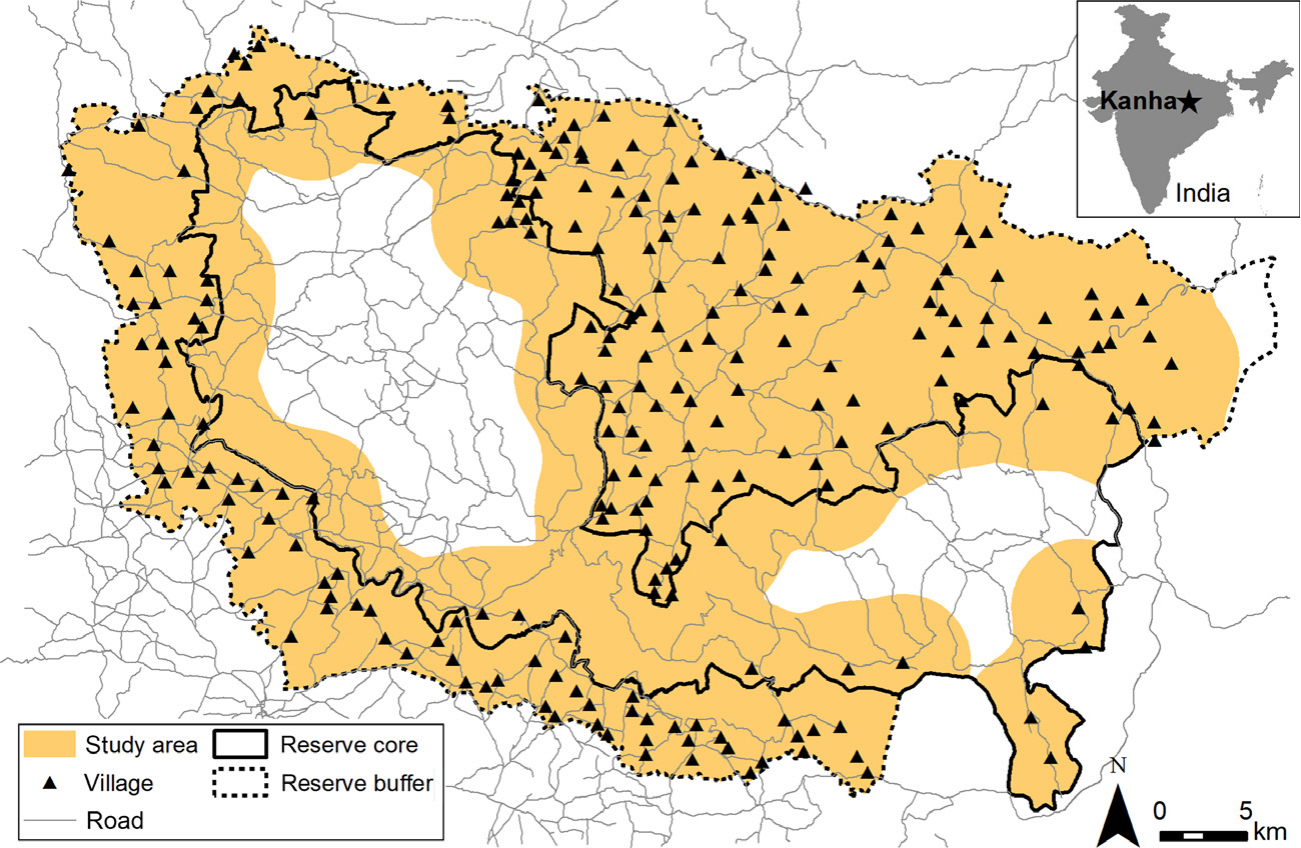
Study area within the core and buffer zones of Kanha Tiger Reserve in Madhya Pradesh, Central India with respect to protected area boundaries, roads, and villages.

The study was conducted in Kanha Tiger Reserve, Madhya Pradesh, central India (Fig.1), where tigers frequently kill and consume domestic cattle (*Bos indicus*), buffalo (*Bubalus bubalis*), pigs (*Sus scrofa*), and goats (*Capra aegagrus hircus*) throughout the 2074 km^2^ protected area. Kanha consists of a 1134 km^2^ multiple-use buffer zone, where human residences and activities such as livestock grazing are allowed, surrounding a 940 km^2^ national park core zone, where human activities are restricted. Only the few villages located inside the core zone are permitted to graze livestock in the core within designated areas around their villages. The core zone is inhabited by 8300 people with 6800 cattle compared to 129,300 people with 85,100 cattle in the buffer (estimates for other livestock were not available; Kanha Tiger Reserve Forest Department 2012).

Tigers and other large carnivores that use ambush hunting attack and kill prey within areas of 9–80 m (Schaller 1967; J. Miller, personal observation), suggesting that carnivore decisions when attacking and killing livestock occur at these fine spatial resolutions. We therefore examined landscape features associated with livestock kills at 20, 100, and 200 m, three spatial grains ranging through and slightly beyond the attack and kill stages of large carnivores using ambush hunting. Our focus at such fine spatial resolutions further offers the kinds of insight necessary to develop spatially informed models of predator–prey interactions at large landscape scales that will enhance conservation and management in human-dominated landscapes (Trainor and Schmitz 2014; Trainor et al. 2014).

We conducted our study in the Kanha Tiger Reserve core and buffer zones where we were confident tiger and livestock species were present. Because data on tiger and prey population occupancy or density were not available at fine spatial resolutions, we built our models based on the informed assumption that tigers and livestock were present throughout the reserve. The Kanha core zone contains a large, stable population of 70 tigers (Jhala et al. 2014) that maintain home range sizes of 10–102 km^2^ (Sharma et al. 2010) and move through the park core, buffer, and the corridors surrounding the protected area (WWF-India 2011; Sharma et al. 2013). Our field observations also revealed that at least 84% of sampled sites contained cattle, buffalo, or goat fecal pellets, suggesting that livestock graze widely throughout many microhabitats in the area. Because livestock freely move without herders for half the year when fields are fallow and graze extensively throughout all accessible vegetation of the protected area, livestock presence was considered uniform throughout the landscape. Cattle tracked with GPS collars (*n* = 6) roamed a maximum of 2.6 km outside of village centers (M. Agarwala, unpublished), and we measured livestock mortalities up to 3.7 km from village centers. We consequentially restricted the study area to within 4 km of village centers, which included the outer ring of the core zone and most of the park multiple-use buffer zone (Fig.1). All kill and random sites were sampled within the study area.

### Identifying kill and random sites

**Figure 2:**
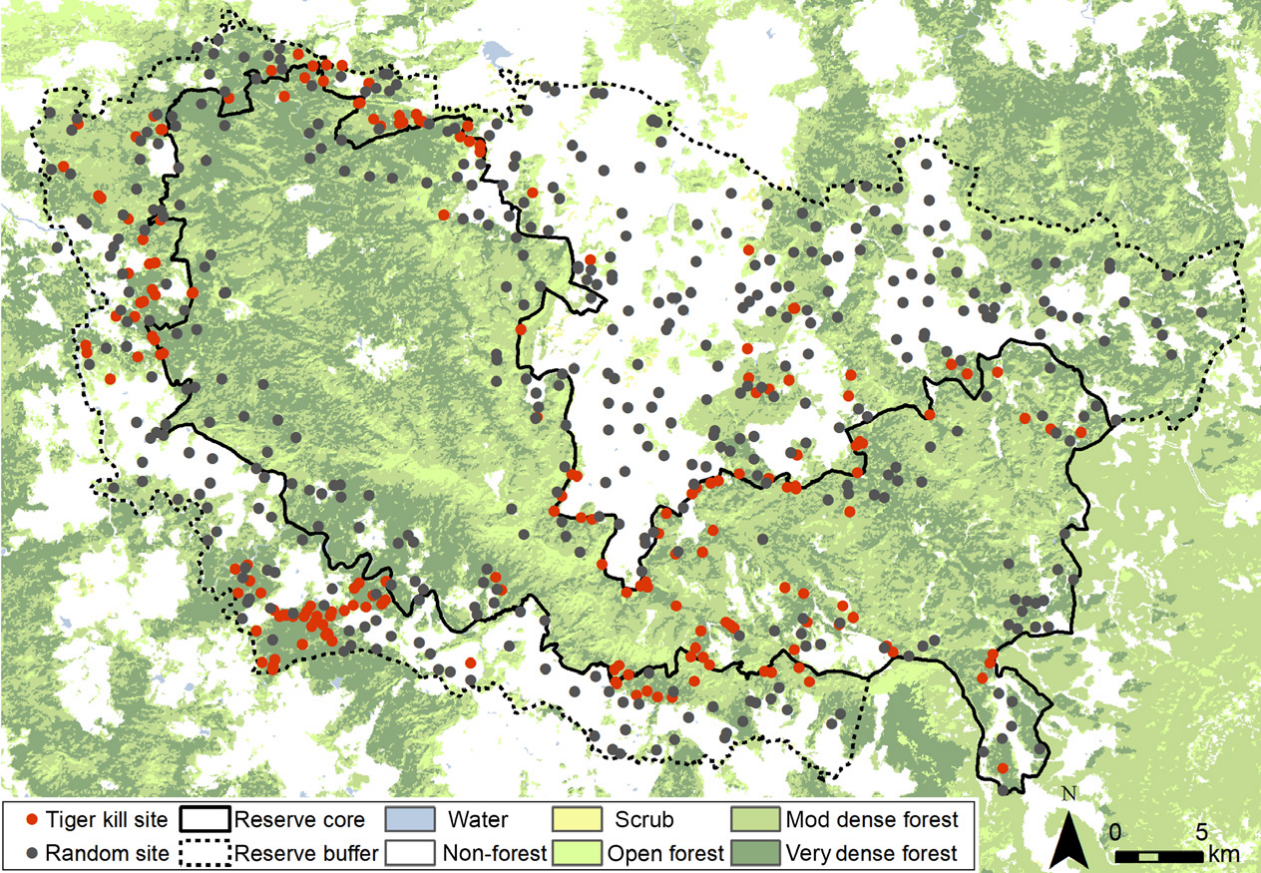
Sampled tiger kill sites and random sites in Kanha Tiger Reserve with respect to protected area boundaries and land-use types.

Between December 2011 and August 2012, we visited livestock kills reported by owners for financial compensation by the Forest Department (Fig.2). Kill sites (where a tiger killed an animal) were distinguished from cache sites (where a tiger dragged and consumed an animal) by drag and scuff marks and trails of blood and hair. Kill site location was measured with an average 5 ± 2 m accuracy using a GPS (Oregon 450, Garmin, KS). Carcasses were identified as tiger kills based on evidence of fresh signs within 50 m of the kill and cache site. Tigers and leopards have notable differences in the size and shapes of their signs (Seidensticker 1976; Karanth and Sunquist 1995), and research technicians were trained to identify signs with high accuracy following the National Tiger Conservation Authority protocol at the Wildlife Institute of India (Jhala et al. 2009). Nonetheless, to ensure accurate predator identity, we classified carnivore signs conservatively and omitted from analysis any kill sites with ambiguous carnivore signs or signs that were located farther than 50 m from the kill site. A total of 90% of all “confirmed” kills were identified using direct sightings of the carnivore (25% of kills), pugmarks (64% of kills), and/or scrapes (2% of kills), which can be clearly distinguished between tigers and leopards (Karanth and Sunquist 1995). Carnivores occasionally killed multiple livestock during a single predation event at one site (*n* = 30 where 2–5 animals died). These cases were treated as a single kill to focus data analysis on units of kill sites (*n* = 1 per kill event) rather than animals killed (*n* = 1 per animal) and to treat data as independent events.

Hunting site selection by tigers was contextualized within the landscape by sampling additional random sites throughout the study area to represent the range of conditions available in the landscape for comparison against kill sites (Manly et al. 2002; Johnson et al. 2006). The location of these sites was determined by random points stratified across a 200-m grid in ArcGIS (v.10.1; ESRI, Redlands, CA), with one point per cell separated by at least 200 m so as not to repeatedly sample. Points were randomly assigned numbers and separated into equal batches by season (#1–200 for winter, #201–400 for summer, #401–600 for monsoon). We surveyed a similar quantity of random sites from each batch each month (27–40 sites) to avoid temporal bias. We visited as many sites as was logistically feasible during the study period to bolster the sample ratio of kill to random sites (Northrup et al. 2013). No wild or domestic prey carcasses were observed at random sites.

### Land-use and human presence variables

Tigers tend to hunt wild prey (e.g., spotted deer, sambar deer, wild pigs) in dense forests near short-grass clearings (Karanth and Sunquist 2000). We therefore expected that the risk of a kill would increase with the density and complexity of vegetation. Many cases of previous livestock kills made by tigers and leopards occurred in villages (Madhusudan 2003) where livestock are abundant (Karanth et al. 2012), yet tigers are also known to avoid areas with intense human activity (Harihar et al. 2007 but see Carter et al. 2012). We predicted that livestock accessibility would show a threshold relationship to villages and roads and peak at an intermediate distance where livestock were vulnerable and humans did not restrict tiger access. We thus expected that predation risk from tigers in dense forests would peak at intermediate distances to humans (and associated livestock).

Landscape variables were measured at each kill and random site for modeling. We collected spatially explicit data on environmental and anthropogenic features known to influence *Felidae* predator ambush attacks on livestock (Table1). These included land-use variables (nonforest [i.e., agriculture], scrubland, moderately dense forest [canopy density of 40–70%; Forest Survey of India 2009] and very dense forest [canopy density of 70% and above]), and human presence variables (roads, villages, and boundary of the park core zone). We did not include topography because previous research in the study area did not find this variable useful for predicting livestock losses (Karanth et al. 2012). Three spatial grains for analysis (20, 100, and 200 m) were tractable units for field measurements (see next section). Landscape variables were converted to raster format and rescaled to these spatial grains using the nearest neighbor resampling (Friedman et al. 1975). At each spatial grain, we calculated the distance to each predictor variable (e.g., distance to land-use types, road, villages, and the core) using the Euclidean distances from each pixel to the nearest feature. All spatial calculations were made using the Spatial Analyst toolset in ArcGIS.

### Vegetation structure variables

Vegetation structure can affect the hunting success of large predators that ambush prey (Table1). We conducted on-the-ground measurements to characterize vegetation structure variables at kill and random sites. These variables were spatially implicit (sampled at specific sites rather than continuously across the landscape) and thus were not included in predation risk RSFs but offered valuable additional insight into the landscape features where livestock are vulnerable to tigers. We measured shrub height, percent shrub cover, shrub patchiness, and visibility in nested 20-m-, 100-m-, and 200-m-diameter circular plots (Fig. S1). Measurements were taken in one central 20-m-diameter subplot and two additional subplots at 40-m intervals along three transects radiating out from the plot center for a total of seven subplots per site. The first transect was selected using a random compass bearing (selected from a list of randomly ordered bearings) and the latter two were placed at 120° intervals to ensure that vegetation across the area was represented. Shrub height was measured to the nearest 0.5 m as the average height of bushy vegetation less than 3 m tall. Percent shrub cover was estimated by eye to the nearest 10% as the percent of ground area covered by shrubs. Shrub patchiness represented the variation of shrub vegetation and was calculated as the standard deviation of shrub cover (100 and 200-m spatial grain only). Visibility was measured as the distance at which dense vegetation or substrate obstructed the outward view of a 1.5-m-tall animal (livestock) and was recorded from the center of the random site in the direction of each transect using a laser rangefinder (RifleHunter 1000; Nikon, Tokyo, Japan).

Habitat structure measurements were averaged among subplots at each spatial grain: measurements from the central subplot (*n* = 1) comprised the 20-m-diameter grain, measurements from the central and interior three subplots (*n* = 4) were averaged for the 100 m grain, and measurements from the central, three interior, and three exterior subplots (*n* = 7) were averaged to calculate the 200-m grain. We calculated the mean visibility along the three transects to find a single visibility value for all spatial grains. Kill and random site averages were then calculated to obtain independent estimates, which we examined for differences at each spatial grain using Mann–Whitney *U*-tests. All statistical analyses were conducted in R (v.2.15.3, R Project Development Team, www.r-project.org).

### Modeling and mapping predation risk

Data were gathered on seven spatially explicit (human presence and land use) biologically meaningful predictor variables identified in the literature and from field observations (Table1). To determine which of these variables were most strongly associated with kills, we built logistic regressions for each spatial grain, using kill and random sites as binary responses of 1 and 0, respectively (Burnham and Anderson 2002; Trainor and Schmitz 2014; Trainor et al. 2014). To avoid collinearity between variables in the model, we calculated Spearman's correlation coefficients for pairs of variables and excluded variables with high correlations (*r*_s_ > 0.6). The distance to nonforest was strongly correlated (*r*_s_ = 0.7) with both the distance to village and the distance to very dense forest at all spatial grains (Table S1–S3) and we therefore excluded this variable. We expected that distances to road, village, and scrubland would have a threshold relationship such that effects might decrease in a nonlinear direction at some distance, and we found that including the quadratic structural form of each predictor lowered the global model AIC by ≥2 (Draper and Smith 1993; Burnham and Anderson 2002). A total of six variables with nine terms were included in models: distances to village, village^2^, road, road^2^, core boundary, scrubland, scrubland^2^, moderately dense forest, and very dense forest. These noncorrelated predictor variables were used to build a global model for the 20, 100, and 200-m spatial grains.

Starting with the global models, we generated and ranked models with all combinations of the biologically meaningful predictor variables based on the corrected Akaike's information criterion (AIC_c_) to account for small sample size (Burnham and Anderson 2002). As no single top model emerged (Akaike weight > 0.90), we then averaged the models to produce final logistic regression models (Table S4). We compared the contribution of each variable in the averaged model using relative importance, which represents the sum of the AIC_c_ weights for each predictor variable over all the included models where the variable appeared (Burnham and Anderson 2002). Relative importance ranges from 0 to 1, with importance values of 1 indicating that the variable made strong contribution to the model. We examined the relationship of each predictor variable to the predicted probability of predation risk while holding other variables constant at their means. Multimodel inference modeling was carried out using the R MuMIN package (K. Barton 2013, http://cran.r-project.org/web/packages/MuMIn/MuMIn.pdf).

To illustrate the spatial patterns of livestock kill probabilities, we used models to map predicted predation risk across Kanha using ArcGIS. Predation risk ranged from 0 to 0.77 and was divided equally into four categories of risk for mapping.

### Model validation

We validated whether models could accurately predict future kills by comparing model predictions against an independent, spatially explicit dataset of 70 livestock killed by tigers. Data on kill sites used for model validation were collected between September 2012 and October 2013, after our original collection of data used to develop and train the models. We located and identified tiger kill sites following similar methods as the original training data, with the added advantage that we set camera traps for 24–48 h at livestock kills to help confirm carnivore species identity (*n* = 16). The GPS coordinates of kills were recorded at the carcass cache site rather than the attack site. We therefore used zonal statistics in ArcMap to extract the maximum risk value within a buffer area around each kill. The buffer was set equal in size to the average drag length (33 m) measured during sampling of our original dataset used to develop the models.

We validated model predictions using randomization (permutation) tests (Edgington 1995). Randomization tests are a powerful way to test for differences among data when the underlying frequency distribution of the data is unknown or likely not to be normal. Such a test examines the probability of obtaining an observed value from a distribution of randomly sampled values. We tested whether the model for each spatial grain designated the validation sites as true “kills” (vs. “no kills”) relative to randomly selected sites. Tests were carried out in several stages (Fig. S2).

First, using our original dataset, we produced a binary map for each model showing the locations where risk was considered high enough for kills to occur. We followed a robust statistical technique used in species distribution modeling (Liu et al. 2013) to identify the threshold value used by each model to classify a risk value as either a “kill” or “no kill”. We calculated the threshold risk value that maximized the sensitivity and specificity (the model's ability to accurately select true “kills” and avoid false “kills”) in the receiver operating characteristic (ROC) curve of each model (Fielding and Bell 1997, Liu et al. 2013). At each spatial grain, we then classified map pixels with risk values less than the model's threshold value as “no kill” and pixels with risk values equal to or greater than the threshold value as “kill”.

We next overlaid the locations of validation kill sites and randomly selected sites onto the pixels of the binary maps. We generated a random distribution by randomly selecting 1000 batches of 70 pixels (equal to the sample size of validation kill sites). We then counted the number of pixels with validation and random sites designated as “kills”. The model for each spatial grain was deemed better than random if the number of sites classified as “kills” in the validation dataset exceeded 95% of the 1000 samples of random sites (95% of the random distribution). *P*-values for randomization tests were calculated as the proportion of random samples equal in value to the observed sample (Edgington 1995), or number of sites classified as “kills” for the validation dataset divided by the number of total random samples (*n* = 1000).

## Results

After excluding sites with unconfirmed or nontiger predators, we analyzed data from 138 tiger kill sites and 439 random sites (Fig.2).

### Model predictions of predation risk

**Figure 3:**
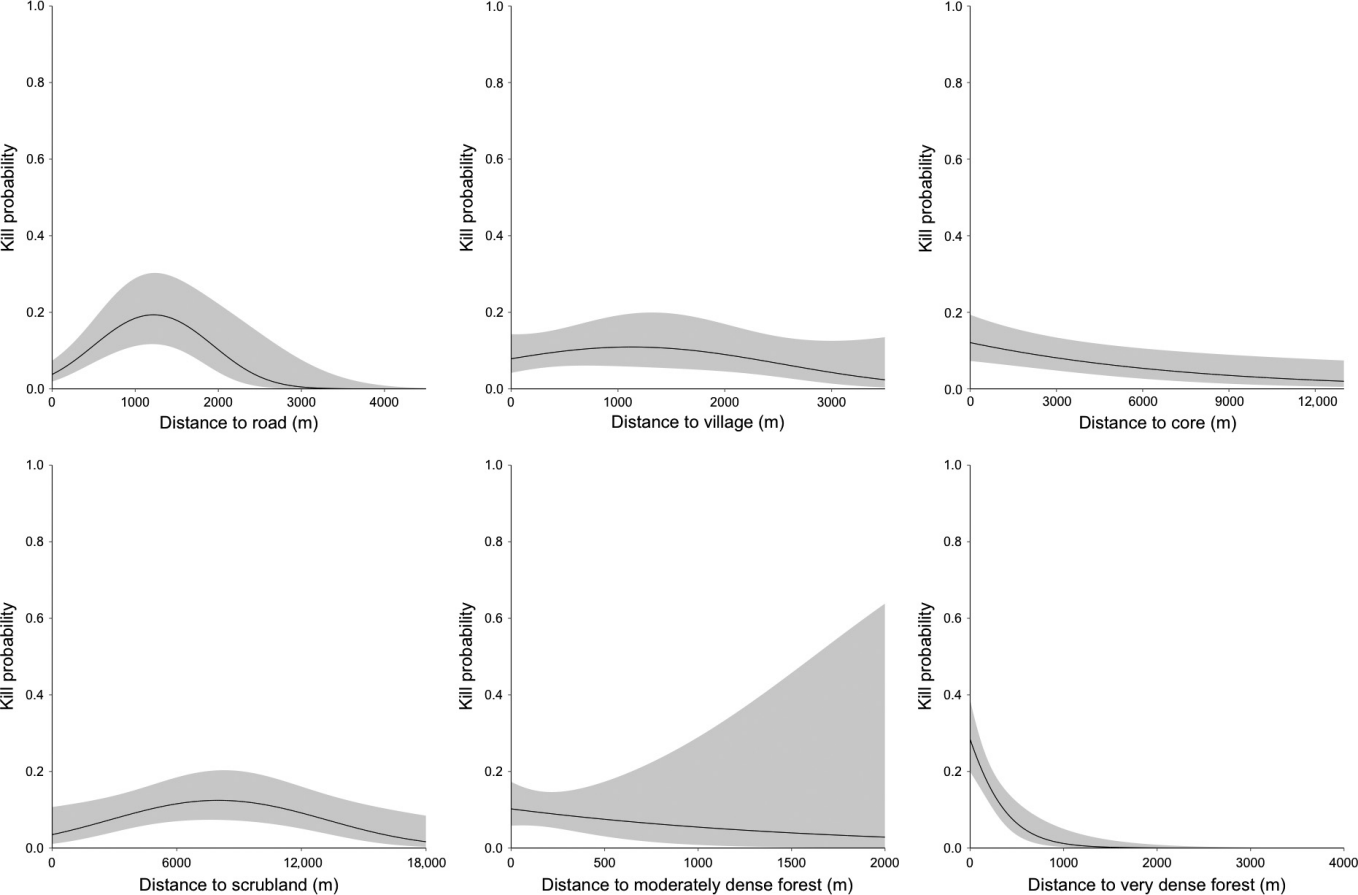
Relationship between each predictor variable and kill probability. The 95% confidence intervals are shown in gray.

Models predicted the probability of a tiger killing a livestock given an encounter between both species. Livestock were most accessible to tigers close to very dense forest (such as near the core zone of the park) and away from roads, villages, and scrubland (Fig.3). As predicted, the risk to livestock increased with closer proximity to very and moderately dense forest and the core zone boundary. Kill probability showed a quadratic relationship to the distance to road, village, and scrubland, with livestock vulnerability increasing at farther distances up to a threshold point and thereafter decreasing. Livestock were most accessible to tigers around 1.2 km from roads, 1.1 km from villages, and 8.0 km from scrubland (Fig.3).

**Figure 4:**
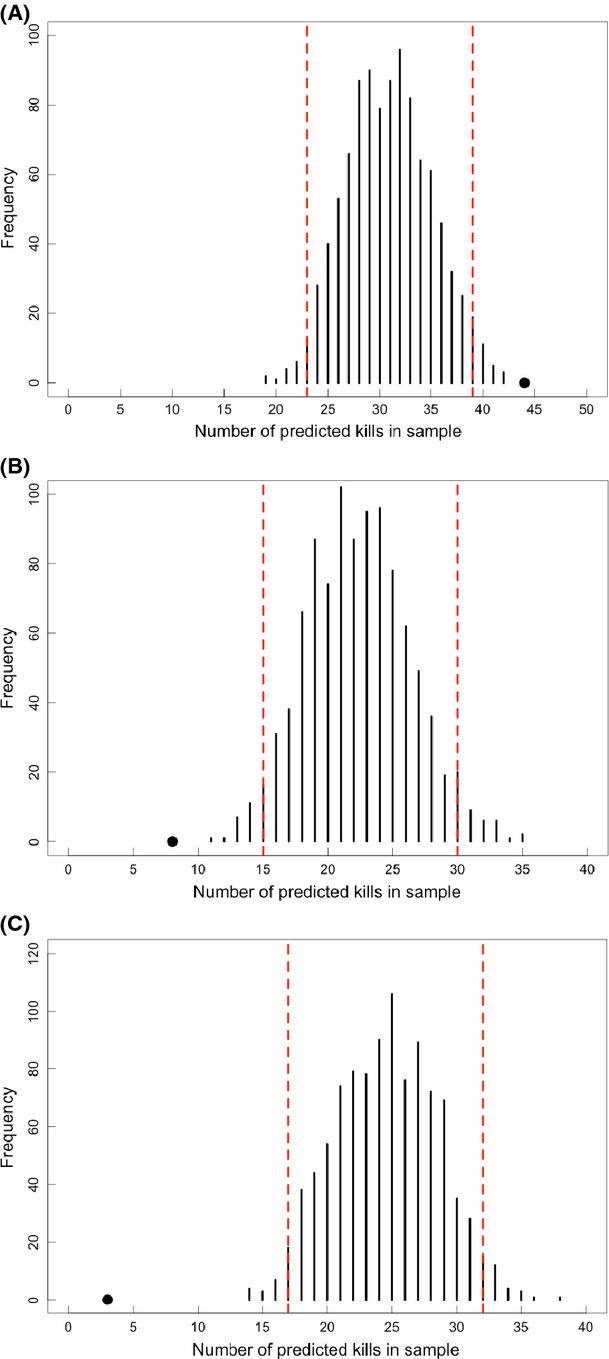
Model validation results for randomization tests using an independent dataset of known tiger kills (*n* = 70) for models at (A) 20-m, (B) 100-m, and (C) 200-m spatial grains. The random distribution (black bars) was calculated by sampling 1000 batches of 70 randomly selected sites from binary predation risk maps designated as “kill” or “no kill” (see Methods for details). Each black bar represents the frequency of random samples (out of 1000) with the given number of random sites designated by the model as “kills”. Dashed red lines bound 95% of the random distribution. Solid points represent the validation dataset and show the number of known tiger kills that were accurately classified by the model as “kills”. Solid points located beyond 95% of the random distribution indicate that predictive performance is significantly better than random.

**Figure 5:**
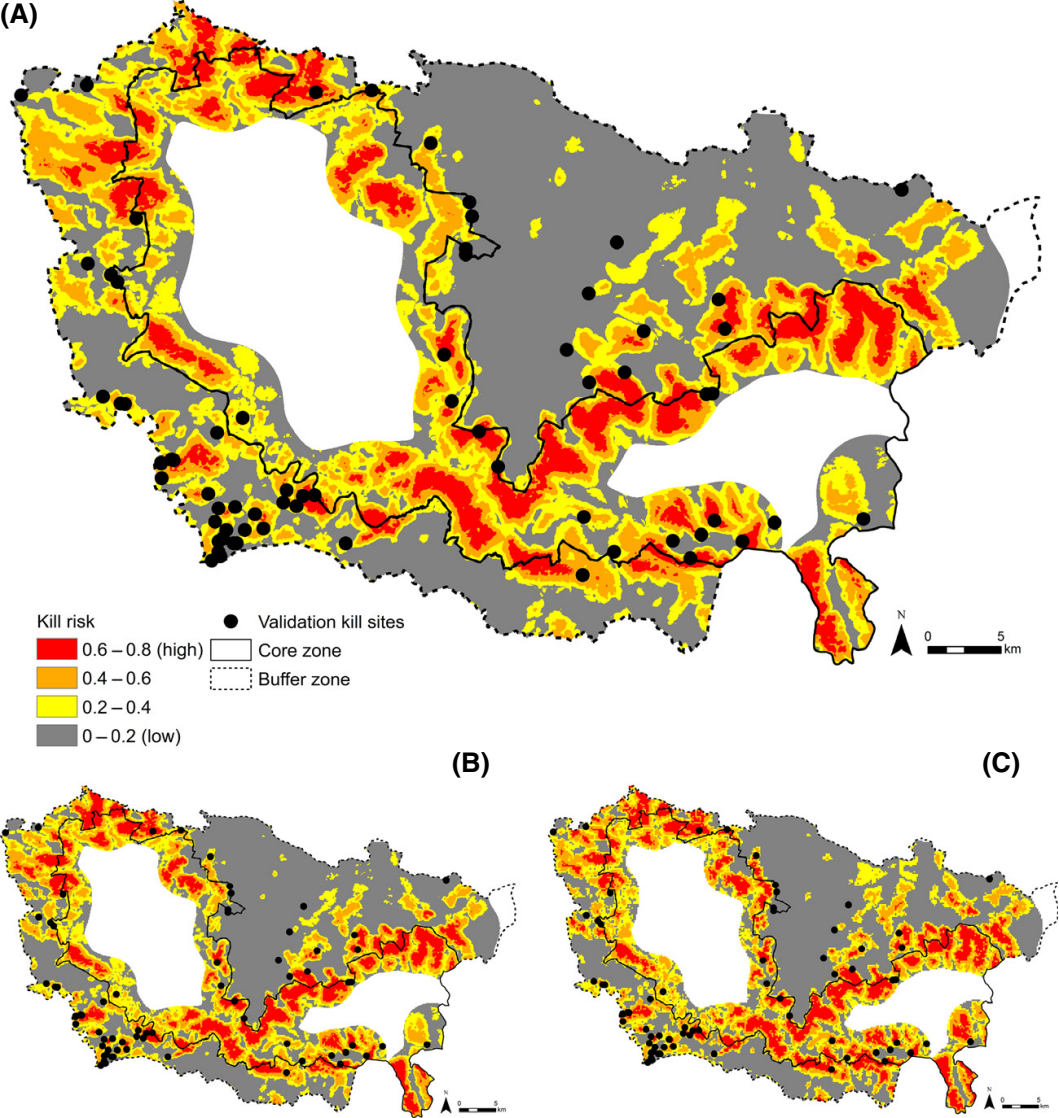
Predicted risk of tiger killing livestock in Kanha Tiger Reserve modeled at spatial grains of (A) 20 m, (B) 100 m, and (C) 200 m. Validation against an independent dataset of known tiger kill sites (solid black circles shown in [A]) indicated strong predictive accuracy at 20 m but not 100 m or 200 m (see Methods for details). White regions represent areas outside the study area that were not modeled.

The contribution of each variable to predictions of predation risk was measured by its relative importance in the model. In the 20 m risk model, which was validated as having the most accurate predictive performance (Figs.4, 5A), distance to road^2^, very dense forest, core, scrub^2^, and village^2^ all ranked ≥0.85 in relative importance and most strongly explained the location of kills (Table2). The relative importance of most variables remained high across spatial grains and only two main variables decreased in importance across spatial grains: distance to moderately dense forest at 20 m and distance to scrubland^2^ at 200 m.

**Table 2:** Predation risk model output at three spatial grains showing the predictor variable relative importance, coefficient (*β*), and standard error (SE) in the final averaged model. Relative importance values range from 0 to 1, with a value of 1 indicating a strong contribution to the model

Predictor variable	Model spatial grain								
	20 m			100 m			200 m		
	Importance	*β*	SE	Importance	*β*	SE	Importance	*β*	SE
Intercept		−2.58	0.71		−2.18	0.77		−0.73	0.60
Distance to village	0.73	8.9E-04	5.5E-04	0.69	8.3E-04	5.6E-04	0.61	6.6E-04	5.9E-04
Distance to village^2^	0.85	−3.4E-07	2.0E-07	0.87	−3.1E-07	2.0E-07	0.84	−2.6E-07	1.8E-07
Distance to road	1.00	3.0E-03	6.6E-04	1.00	2.8E-03	6.4E-04	1.00	2.8E-03	6.2E-04
Distance to road^2^	1.00	−1.2E-06	3.3E-07	1.00	−1.2E-06	3.3E-07	1.00	−1.1E-06	3.2E-07
Distance to core	0.99	−1.5E-04	5.4E-05	1.00	−1.5E-04	5.4E-05	0.96	−1.3E-04	5.0E-05
Distance to scrub	0.95	3.6E-04	1.3E-04	0.93	3.7E-04	1.3E-04	0.40	9.3E-05	1.3E-04
Distance to scrub^2^	0.97	−2.2E-08	8.1E-09	0.95	−2.2E-08	8.3E-09	0.47	−5.9E-09	6.2E-09
Distance to moderately dense forest	0.44	−1.5E-03	1.2E-03	0.91	−2.5E-03	1.0E-03	1.00	−2.9E-03	7.7E-04
Distance to very dense forest	1.00	−3.5E-03	8.7E-04	1.00	−3.1E-03	7.9E-04	1.00	−2.8E-03	6.3E-04

### Model validation

Using threshold risk values from the ROC, we designated “kills” or “no kills” if they, respectively, fell above or below 0.26 for 20 m, 0.39 for 100 m, and 0.39 for 200 m. Randomization tests revealed that predictions for the validation data sites exceeded the 95% limits of the random data sites, that is, the risk models performed differently than random (Fig.4). However, only the model for the finest grain (20 m) predicted kills that far exceeded the 95% distribution (Fig.4A). Risk predictions for validation sites from the coarser grain models (100 and 200 m) fell on the low side of the distribution (Fig.4B and C). The 20 m model accurately identified 65% of validation sites (44 of 70 known kill sites) as kills, which is greater than would be expected by random chance (*P *=* *0.01). The 100 and 200 m models had substantially weaker predictive accuracy, identifying only 11% and 4% of validation kills as kills.

### Vegetation structure

Measurements of vegetation structure revealed that undergrowth complexity and heterogeneity differed significantly between kill sites and random sites. Shrub height, percent shrub cover, and shrub patchiness were all higher in the vicinity of livestock kills than random sites, generating less visibility at kills (*P *<* *0.001; Table3).

**Table 3:** Mann–Whitney *U*-test statistics showing the test coefficient (*W*) and *P*-value (*P*) for vegetation structure predictor variables between kill sites and random control sites at the three spatial grains. All values are statistically significant (*P *<* *0.05)

Spatial grain (m)	Predictor variable	*W*	*P*
20	Visibility	40571.0	1.8E-09
	Shrub cover	19463.5	1.9E-10
	Shrub height	17430.0	1.7E-14
100	Visibility	40571.0	1.8E-09
	Shrub cover	19008.0	3.9E-11
	Shrub height	17008.5	6.9E-15
	Shrub patchiness	18266.0	1.7E-12
200	Visibility	40571.0	1.8E-09
	Shrub cover	18778.5	1.6E-11
	Shrub height	16952.0	5.3E-15
	Shrub patchiness	18610.5	7.8E-12

### Spatial patterns of predation risk

Predation risk maps offered visual insight into the spatial distribution of predicted livestock depredations. Maps showed greater risk within forest patches and lower risk around more open vegetation (scrublands and agricultural fields), roads, and villages (Fig.5). Models predicted that 8–11% (169–232 km^2^, depending on spatial grain) of the Kanha Tiger Reserve fell into the highest risk category (0.61–0.80).

## Discussion

Past investigations of tiger hunting behavior and ecology have largely focused on natural prey (e.g., Karanth and Sunquist 2000; Karanth et al. 2004; Simcharoen et al. 2014). The few studies that have examined attacks on livestock utilized household surveys to deduce spatial patterns of conflicts at the village level (Madhusudan 2003; Karanth et al. 2012) rather than directly examining livestock kill sites. Examining the biophysical attributes at and around kill sites, as we do here, offers key complementary insight needed to directly explain the landscape features associated with carnivore hunting that influence livestock vulnerability to predation.

Our analyses revealed that land-use and human presence variables modeled at a fine spatial grain predicted the spatial patterns of livestock kills by large carnivores that ambush prey, such as tigers. Livestock were more accessible near dense, patchy forests with tall scrub, and poor visibility than near open vegetation. The probability of a kill increased with greater distance from roads and villages until animals were around 1 km from infrastructure, which may represent a threshold distance where livestock can conveniently access quality browse or where tigers can access livestock uninhibited by human activity. The kill probability response was constantly low for villages compared to other landscape attributes (Fig.3), demonstrating tiger aversion to human presence and the lower likeliness of an attack on livestock within village areas. Maps illustrated a region of high risk ringing the inside of the park, illustrating the heightened chance of attacks on livestock grazing within the protected core zone.

### Relevance of spatial grain for predicting kills

All variables contributed strongly to models across all spatial grains except distance to scrubland^2^ and moderately dense forest, which decreased in relative importance in the models at the coarsest and finest grains, respectively. These exceptions are consistent with carnivore behavior because the presence of forest is more likely to impact broadscale decisions made during the search phase of hunting, whereas the presence of scrubs more likely plays a greater role in fine-scale decisions made at the moment of a kill (i.e., as a determinant of prey accessibility). The shifts in variable importance suggest that tigers alter their use of landscape features when interacting with prey at different spatial resolutions and underscore the importance of modeling predation risk at a grain that best represents the species interaction of interest for management and conservation goals.

Using past kill events to anticipate and avoid future human–carnivore conflict is one such priority for management and conservation (Treves et al. 2011). For our analysis, we used previous livestock kill sites to predict future kills. External validation against an independent dataset of kills found that the finest resolution model (20 m) accuracy predicted the majority of kills, whereas the coarser spatial grains (100 and 200 m) did not. Many of the landscape variables included in models were highly spatially heterogeneous at the fine scale. As the spatial grain increased, this heterogeneity reduced and the coarser grain models obscured the accuracy of predictions. Representative of many large carnivores that ambush prey, tigers tend to attack and kill prey over a small area (Schaller 1967), necessitating fine-resolution models that can capture the landscape features associated with species decisions made during the attack and kill. This spatial grain contrasts with the resolution used by other predation risk studies modeling the risk of livestock depredation, which span 1–25 km (Kaartinen et al. 2009; Marucco and McIntire 2010; Zarco-González et al. 2013; Soh et al. 2014). The coarse scales used in these studies likely capture landscape features important in a carnivore's search for prey across broad spatial grains (Gorini et al. 2012) and thereby represent a range of general predator–prey encounters (Hebblewhite et al. 2005), which may include kills but may not necessarily pinpoint prey accessibility. Many free datasets of environmental satellite data are available at different spatial grains and we emphasize the need to carefully consider how the resolution and type of interaction data (e.g., encounters versus kill sites) may influence outcomes prior to modeling (Hebblewhite et al. 2005; Hilborn et al. 2012). Our results in particular demonstrate that fine spatial grains <100 m represent species decisions made during the attack and kill for large carnivores that ambush prey. Coarser scales are likely more appropriate for active carnivores that attack and kill over larger areas (Kaartinen et al. 2009; Davie et al. 2014).

### Landscape features associated with kills

Livestock were most vulnerable to tigers within the core zone boundary of the park, where very dense forests located somewhat away from roads and villages provide ideal hunting grounds for tigers. Grazing livestock within the core of Kanha Tiger Reserve is not permitted for most villages in an effort to preserve forest quality and prevent attacks on livestock and people, and predation risk maps serve as a visual reminder of the high risks of grazing inside the core. Our study suggests that herders aiming to reduce livestock losses should prioritize grazing in scrubland and open vegetation near roads and villages but outside of dense forests. The risk of carnivores killing livestock is merely one of many factors affecting decisions about where to graze livestock; thus, our findings offer simple and necessary guidance to assist natural resource managers and livestock owners in this complex process.

In addition to the land-use and human presence variables featured in risk models, vegetation structure variables also differed significantly between kill and random sites. Vegetation complexity was markedly higher and overall visibility lower at kill sites. This is expected for large carnivores that ambush prey, which utilize dense vegetation to inconspicuously attack prey (Table1). We were unable to include vegetation structure in our spatial models because spatially explicit data were not available to represent the entire Kanha landscape, yet including such information might have strengthened the predictive accuracy of our models. We encourage future studies to take advantage of advancing techniques for modeling the structural complexity of vegetation based on remotely sensed data (Estes et al. 2010).

Several modeling constraints should be noted when considering the generalizability of our findings. First, by basing tiger predation risk models on kill sites rather than predator–prey encounters more inclusively, this study offers conservative predictions about the probability of a tiger killing a livestock given an encounter between the two species (Hebblewhite et al. 2005; Gorini et al. 2012). Second, as species occupancy data with a spatial resolution of less than 100 km^2^ are currently not available for our study region, we limited the study to the protected area where large populations of tigers and livestock prey are known. The risk model would thus need to account for predator and prey presence and resource selection if applied to areas outside the protected area where species presence is more uncertain. Nonetheless, our findings on the role of spatial resolution in modeling predation risk have applications for systems with human–carnivore conflict worldwide. Finally, we recognize that our dataset may not account for unreported livestock owned by the minority of villagers in the park not reporting losses to the Forest Department (Karanth et al. 2012).

### Broader implications for conservation

Maps generated by spatial predation risk models offer powerful visual guides for communicating patterns of carnivore predation risk to stakeholders from diverse educational and cultural backgrounds (Rambaldi et al. 2006; Brown and Raymond 2007). Spatial records of kills are routinely collected in many regions (Woodroffe et al. 2005; Gorini et al. 2012) and can be paired with modeling to regularly produce up-to-date risk maps to track conflict hotspots in near-real time. Predation risk models and maps could be useful for numerous conservation applications at various scales, such as policymakers allocating financial resources for livestock insurance or compensation schemes, managers prioritizing areas for land-use zoning, and livestock herders selecting routes for grazing. We emphasize the importance of collecting geospatial data associated with predator–prey interactions and encourage practitioners to incorporate risk modeling into the infrastructure of conservation programs.

As human populations continue to grow and compete with carnivores for natural resources, innovative tools such as predation risk models are increasingly necessary for planning compatible land use and coexistence at landscape scales (Walston et al. 2010; Treves et al. 2011). As model outcomes and biological insights about predator–prey interactions are sensitive to the spatial resolution of analysis (Hebblewhite et al. 2005; Hilborn et al. 2012), we recommend developing predation risk maps at the finest spatial grain that meets specific conservation or management objectives. When produced with the appropriate spatial resolution, predation risk models can produce strong quantitative predictions that facilitate science-informed land use and management.

## Supporting Information

**Figure S1.** Sampling design for measuring vegetation structure. **Figure S2.** Workflow of the model validation methods. **Table S1.** Correlation matrices for spatially explicit variables at 20 m. **Table S2.** Correlation matrices for spatially explicit variables at 100 m. **Table S3.** Correlation matrices for spatially explicit variables at 200 m. **Table S4.** Predation risk models averaged to produce the final model.
