# Supplementary material for: Landscape-scale accessibility of livestock to tigers: implications of spatial grain for modeling predation risk to mitigate human–carnivore conflict
Source: Ecol Evol. 2015 Mar 2;5(6):1354–67. doi: 10.1002/ece3.1440 (PMC4377277; doi:10.1002/ece3.1440)
Supplement: Supplementary file 1 [file ece30005-1354-sd1.docx]

**Supporting Information**

Landscape-scale accessibility of livestock to tigers: implications of spatial grains for modeling predation risk to mitigate human-carnivore conflict

J. R. B. Miller, Y. V. Jhala, J. Jena & O. J. Schmitz

**Contents**

Figure S1. Sampling design for measuring vegetation structure.

Figure S2. Workflow of the model validation methods.

Table S1. Correlation matrices for spatially explicit variables at 20 m.

Table S2. Correlation matrices for spatially explicit variables at 100 m.

Table S3. Correlation matrices for spatially explicit variables at 200 m.

Table S4. Predation risk models averaged to produce the final model.

**Predictor variables and abbreviations in Supporting Information**

| Variable (unit) | Abbreviation |
| --- | --- |
| Distance to road (m) | disrd |
| Distance to village (m) | disvil |
| Distance to core (m) | discore |
| Distance to non-forest (m) | disNF |
| Distance to moderately dense forest (m) | dismodenfor |
| Distance to very dense forest (m) | disvrydenfor |
| Distance to scrubland (m) | disscrub |
| Visibility (m) | vis |
| Shrub height (m) | shrubhght |
| Shrub cover (%) | shrubcvr |
| Shrub patchiness (%) | patchiness |


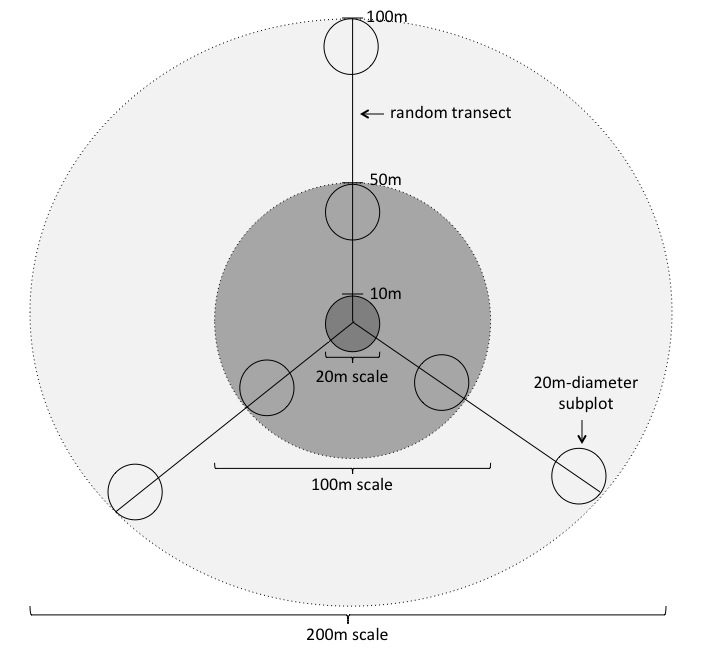


Figure S1. Sampling design for measuring vegetation structure at kill and random sites.


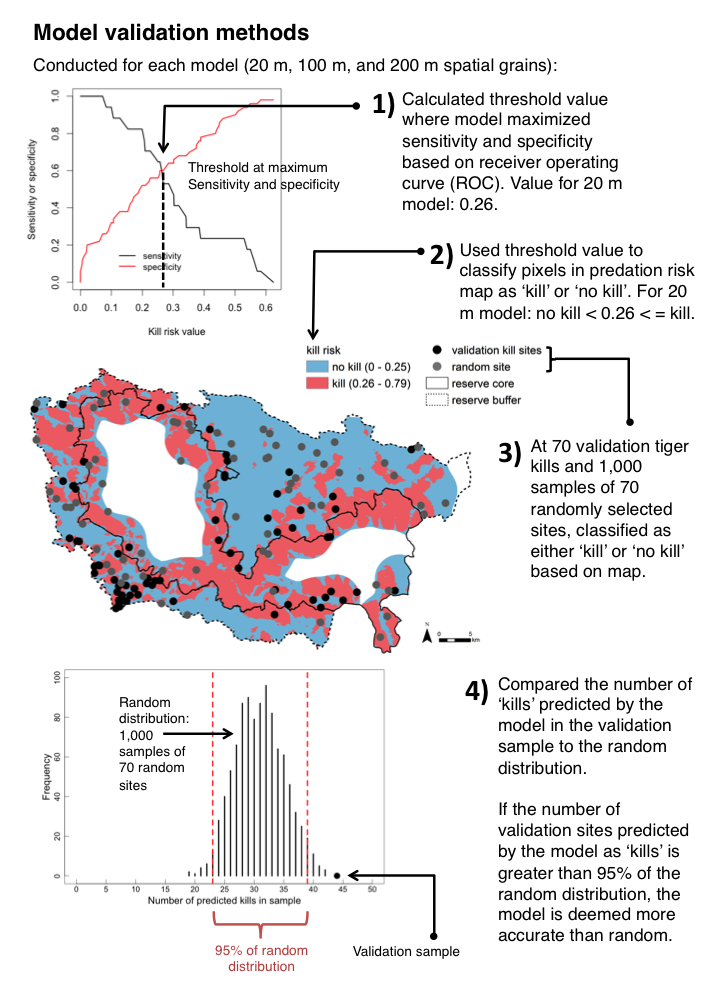


Figure S2. Workflow of the model validation process.

Table S1. Correlation matrices for spatially explicit variables at 20 m.

|  | Correlation strength | | | | | | |
| --- | --- | --- | --- | --- | --- | --- | --- |
| Variable | disvil | disrd | discore | FSIdisNF | FSIdisS | FSIdisMDF | FSIdisVDF |
| disvil | 1.0 | 0.2 | -0.3 | 0.7 | 0.3 | -0.4 | -0.5 |
| disrd | 0.2 | 1.0 | 0.0 | 0.1 | 0.0 | -0.1 | -0.1 |
| discore | -0.3 | 0.0 | 1.0 | -0.3 | -0.4 | 0.2 | 0.3 |
| FSIdisNF | 0.7 | 0.1 | -0.3 | 1.0 | 0.3 | -0.6 | -0.7 |
| FSIdisS | 0.3 | 0.0 | -0.4 | 1.0 | 1.0 | -0.2 | -0.5 |
| FSIdisMDF | -0.4 | -0.1 | 0.2 | 0.3 | -0.2 | 1.0 | 0.4 |
| FSIdisVDF | -0.5 | -0.1 | 0.3 | -0.6 | -0.5 | 0.4 | 1.0 |

Table S2. Correlation matrices for spatially explicit variables at 100 m.

|  | Correlation strength | | | | | | |
| --- | --- | --- | --- | --- | --- | --- | --- |
| Variable | disvil | disrd | discore | FSIdisNF | FSIdisS | FSIdisMDF | FSIdisVDF |
| disvil | 1.0 | 0.2 | -0.3 | 0.7 | 0.3 | -0.4 | -0.5 |
| disrd | 0.2 | 1.0 | 0.0 | 0.1 | 0.0 | -0.1 | -0.2 |
| discore | -0.3 | 0.0 | 1.0 | -0.3 | -0.4 | 0.2 | 0.3 |
| FSIdisNF | 0.7 | 0.1 | -0.3 | 1.0 | 0.3 | -0.6 | -0.7 |
| FSIdisS | 0.3 | 0.0 | -0.4 | 1.0 | 1.0 | -0.2 | -0.5 |
| FSIdisMDF | -0.4 | -0.1 | 0.2 | 0.3 | -0.2 | 1.0 | 0.4 |
| FSIdisVDF | -0.5 | -0.2 | 0.3 | -0.6 | -0.5 | 0.4 | 1.0 |

Table S3. Correlation matrices for spatially explicit variables at 200 m.

|  | Correlation strength | | | | | | |
| --- | --- | --- | --- | --- | --- | --- | --- |
| Variable | disvil | disrd | discore | FSIdisNF | FSIdisS | FSIdisMDF | FSIdisVDF |
| disvil | 1.0 | 0.2 | -0.3 | 0.7 | 0.3 | -0.4 | -0.5 |
| disrd | 0.2 | 1.0 | 0.0 | 0.1 | 0.0 | -0.1 | -0.2 |
| discore | -0.3 | 0.0 | 1.0 | -0.3 | -0.3 | 0.2 | 0.3 |
| FSIdisNF | 0.7 | 0.1 | -0.3 | 1.0 | 0.3 | -0.5 | -0.7 |
| FSIdisS | 0.3 | 0.0 | -0.3 | 1.0 | 1.0 | -0.2 | -0.5 |
| FSIdisMDF | -0.4 | -0.1 | 0.2 | 0.3 | -0.2 | 1.0 | 0.3 |
| FSIdisVDF | -0.5 | -0.2 | 0.3 | -0.5 | -0.5 | 0.3 | 1.0 |

Table S4. Tiger predation risk models averaged to produce the final model (models shown with ΔAIC_c_<7). Statistics shown are the degrees of freedom (df), log likelihood (logLik), Akaike Information Criterion (AICc), change in AICc (delta) and model weight (weight).

| **Spatial**  **grain (m)** | **Predictor variable** | | | | | | | | | | **Statistics** | | | | |
| --- | --- | --- | --- | --- | --- | --- | --- | --- | --- | --- | --- | --- | --- | --- | --- |
|  | Intercept | discore | disrd | disrd_2 | disvil | disvil_2 | FSIdisMDF | FSIdisS | FSIdisS_2 | FSIdisVDF | df | logLik | AICc | delta | weight |
| 20 | -2.83 | -1.5E-04 | 2.9E-03 | -1.2E-06 | 1.0E-03 | -4.1E-07 | NA | 3.6E-04 | -2.3E-08 | -3.5E-03 | 9 | -245.37 | 509.05 | 0.00 | 0.33 |
| 20 | -2.78 | -1.5E-04 | 3.0E-03 | -1.2E-06 | 9.6E-04 | -4.0E-07 | -1.5E-03 | 3.7E-04 | -2.3E-08 | -3.1E-03 | 10 | -244.59 | 509.56 | 0.51 | 0.26 |
| 20 | -2.25 | -1.5E-04 | 3.0E-03 | -1.2E-06 | NA | -9.3E-08 | -1.7E-03 | 3.6E-04 | -2.2E-08 | -3.5E-03 | 9 | -246.71 | 511.74 | 2.68 | 0.09 |
| 20 | -2.28 | -1.5E-04 | 3.0E-03 | -1.2E-06 | NA | -8.6E-08 | NA | 3.5E-04 | -2.2E-08 | -4.0E-03 | 8 | -247.76 | 511.77 | 2.71 | 0.09 |
| 20 | -2.39 | -1.4E-04 | 3.0E-03 | -1.2E-06 | NA | NA | NA | 3.4E-04 | -2.2E-08 | -3.9E-03 | 7 | -249.28 | 512.76 | 3.71 | 0.05 |
| 20 | -2.37 | -1.4E-04 | 3.0E-03 | -1.2E-06 | NA | NA | -1.5E-03 | 3.5E-04 | -2.2E-08 | -3.5E-03 | 8 | -248.51 | 513.28 | 4.22 | 0.04 |
| 20 | -2.25 | -1.5E-04 | 3.0E-03 | -1.2E-06 | -1.4E-04 | NA | NA | 3.4E-04 | -2.2E-08 | -4.0E-03 | 8 | -248.84 | 513.93 | 4.87 | 0.03 |
| 20 | -2.21 | -1.5E-04 | 3.1E-03 | -1.2E-06 | -1.6E-04 | NA | -1.6E-03 | 3.5E-04 | -2.2E-08 | -3.6E-03 | 9 | -247.87 | 514.06 | 5.01 | 0.03 |
| 20 | -1.68 | -1.4E-04 | 2.9E-03 | -1.2E-06 | 9.7E-04 | -4.0E-07 | NA | NA | NA | -3.5E-03 | 7 | -250.28 | 514.75 | 5.69 | 0.02 |
| 20 | -1.45 | -1.5E-04 | 2.9E-03 | -1.2E-06 | 9.4E-04 | -3.9E-07 | NA | NA | -2.3E-09 | -3.7E-03 | 8 | -249.46 | 515.17 | 6.12 | 0.02 |
| 20 | -1.56 | -1.4E-04 | 2.9E-03 | -1.2E-06 | 9.2E-04 | -3.9E-07 | -1.4E-03 | NA | NA | -3.2E-03 | 8 | -249.55 | 515.35 | 6.29 | 0.01 |
| 20 | -3.22 | NA | 3.0E-03 | -1.2E-06 | 1.0E-03 | -4.0E-07 | NA | 3.7E-04 | -2.2E-08 | -3.6E-03 | 8 | -249.77 | 515.79 | 6.73 | 0.01 |
| 100 | -2.45 | -1.5E-04 | 2.8E-03 | -1.2E-06 | 9.2E-04 | -4.0E-07 | -2.5E-03 | 3.7E-04 | -2.3E-08 | -2.9E-03 | 10 | -242.72 | 505.82 | 0.00 | 0.47 |
| 100 | -1.90 | -1.6E-04 | 2.9E-03 | -1.2E-06 | NA | -1.0E-07 | -2.7E-03 | 3.6E-04 | -2.2E-08 | -3.3E-03 | 9 | -244.61 | 507.54 | 1.72 | 0.20 |
| 100 | -2.70 | -1.5E-04 | 2.8E-03 | -1.2E-06 | 1.0E-03 | -4.2E-07 | NA | 3.6E-04 | -2.3E-08 | -3.2E-03 | 9 | -245.73 | 509.78 | 3.96 | 0.06 |
| 100 | -2.05 | -1.4E-04 | 2.9E-03 | -1.2E-06 | NA | NA | -2.4E-03 | 3.5E-04 | -2.2E-08 | -3.2E-03 | 8 | -246.83 | 509.91 | 4.09 | 0.06 |
| 100 | -1.84 | -1.5E-04 | 2.9E-03 | -1.2E-06 | -2.0E-04 | NA | -2.6E-03 | 3.5E-04 | -2.2E-08 | -3.4E-03 | 9 | -245.88 | 510.07 | 4.25 | 0.06 |
| 100 | -1.18 | -1.4E-04 | 2.8E-03 | -1.1E-06 | 8.7E-04 | -3.8E-07 | -2.5E-03 | NA | NA | -3.1E-03 | 8 | -247.52 | 511.28 | 5.46 | 0.03 |
| 100 | -1.00 | -1.5E-04 | 2.7E-03 | -1.1E-06 | 8.5E-04 | -3.7E-07 | -2.4E-03 | NA | -1.9E-09 | -3.2E-03 | 9 | -246.93 | 512.17 | 6.35 | 0.02 |
| 100 | -2.09 | -1.5E-04 | 2.9E-03 | -1.2E-06 | NA | -8.8E-08 | NA | 3.5E-04 | -2.2E-08 | -3.7E-03 | 8 | -248.19 | 512.63 | 6.80 | 0.02 |
| 100 | -0.72 | -1.4E-04 | 2.8E-03 | -1.1E-06 | NA | -1.0E-07 | -2.7E-03 | NA | NA | -3.4E-03 | 7 | -249.23 | 512.66 | 6.84 | 0.02 |
| 200 | -0.90 | -1.3E-04 | 2.8E-03 | -1.1E-06 | 8.3E-04 | -3.6E-07 | -2.8E-03 | NA | NA | -2.7E-03 | 8 | -244.55 | 505.36 | 0.00 | 0.18 |
| 200 | -1.39 | -1.3E-04 | 2.8E-03 | -1.1E-06 | 8.0E-04 | -3.5E-07 | -2.9E-03 | 1.7E-04 | -1.0E-08 | -2.7E-03 | 10 | -242.94 | 506.27 | 0.91 | 0.11 |
| 200 | -0.71 | -1.3E-04 | 2.7E-03 | -1.1E-06 | 8.1E-04 | -3.5E-07 | -2.8E-03 | NA | -1.5E-09 | -2.8E-03 | 9 | -244.00 | 506.33 | 0.97 | 0.11 |
| 200 | -0.42 | -1.3E-04 | 2.8E-03 | -1.1E-06 | NA | -1.1E-07 | -3.0E-03 | NA | NA | -2.9E-03 | 7 | -246.09 | 506.37 | 1.01 | 0.11 |
| 200 | -0.71 | -1.3E-04 | 2.7E-03 | -1.1E-06 | 8.2E-04 | -3.6E-07 | -2.8E-03 | -1.9E-05 | NA | -2.7E-03 | 9 | -244.34 | 507.00 | 1.64 | 0.08 |
| 200 | -0.91 | -1.3E-04 | 2.8E-03 | -1.1E-06 | NA | -1.1E-07 | -3.0E-03 | 1.7E-04 | -1.0E-08 | -3.0E-03 | 9 | -244.35 | 507.02 | 1.67 | 0.08 |
| 200 | -0.22 | -1.3E-04 | 2.8E-03 | -1.1E-06 | NA | -1.0E-07 | -3.0E-03 | NA | -1.7E-09 | -3.0E-03 | 8 | -245.44 | 507.13 | 1.77 | 0.07 |
| 200 | -0.21 | -1.3E-04 | 2.8E-03 | -1.1E-06 | NA | -1.0E-07 | -3.0E-03 | -2.1E-05 | NA | -3.0E-03 | 8 | -245.81 | 507.88 | 2.52 | 0.05 |
| 200 | -0.36 | -1.2E-04 | 2.8E-03 | -1.1E-06 | -2.4E-04 | NA | -3.0E-03 | NA | NA | -2.9E-03 | 7 | -247.39 | 508.98 | 3.62 | 0.03 |
| 200 | -0.16 | -1.3E-04 | 2.8E-03 | -1.1E-06 | -2.3E-04 | NA | -3.0E-03 | NA | -1.8E-09 | -3.1E-03 | 8 | -246.65 | 509.55 | 4.20 | 0.02 |
| 200 | -0.83 | -1.3E-04 | 2.9E-03 | -1.1E-06 | -2.4E-04 | NA | -3.0E-03 | 1.7E-04 | -1.0E-08 | -3.0E-03 | 9 | -245.62 | 509.56 | 4.20 | 0.02 |
| 200 | -0.69 | -1.1E-04 | 2.8E-03 | -1.1E-06 | NA | NA | -2.8E-03 | NA | NA | -2.7E-03 | 6 | -248.73 | 509.61 | 4.26 | 0.02 |
| 200 | -0.46 | -1.2E-04 | 2.8E-03 | -1.1E-06 | NA | NA | -2.8E-03 | NA | -1.9E-09 | -2.9E-03 | 7 | -247.87 | 509.94 | 4.58 | 0.02 |
| 200 | -1.09 | -1.2E-04 | 2.8E-03 | -1.1E-06 | NA | NA | -2.9E-03 | 1.5E-04 | -9.7E-09 | -2.8E-03 | 8 | -246.98 | 510.22 | 4.86 | 0.02 |
| 200 | -0.13 | -1.3E-04 | 2.8E-03 | -1.1E-06 | -2.3E-04 | NA | -3.0E-03 | -2.3E-05 | NA | -3.0E-03 | 8 | -247.05 | 510.35 | 4.99 | 0.01 |
| 200 | -1.12 | NA | 2.8E-03 | -1.1E-06 | 8.3E-04 | -3.4E-07 | -2.9E-03 | NA | NA | -2.8E-03 | 7 | -248.15 | 510.50 | 5.14 | 0.01 |
| 200 | -0.42 | -1.1E-04 | 2.8E-03 | -1.1E-06 | NA | NA | -2.8E-03 | -2.6E-05 | NA | -2.8E-03 | 7 | -248.29 | 510.78 | 5.42 | 0.01 |
| 200 | -0.64 | NA | 2.9E-03 | -1.1E-06 | NA | -8.6E-08 | -3.0E-03 | NA | NA | -3.1E-03 | 6 | -249.70 | 511.54 | 6.18 | 0.01 |
| 200 | -1.75 | NA | 2.8E-03 | -1.2E-06 | 8.0E-04 | -3.4E-07 | -2.9E-03 | 1.8E-04 | -9.9E-09 | -2.8E-03 | 9 | -246.75 | 511.82 | 6.46 | 0.01 |
| 200 | -1.03 | NA | 2.8E-03 | -1.1E-06 | 8.1E-04 | -3.3E-07 | -2.9E-03 | NA | -8.0E-10 | -2.9E-03 | 8 | -248.00 | 512.25 | 6.89 | 0.01 |

‘NA’ indicates variable was not included in the model.
